# Structural manipulation and tailoring of dielectric properties in SrTi_1−x_Fe_x_Ta_x_O_3_ perovskites: Design of new lead free relaxors

**DOI:** 10.1038/srep23400

**Published:** 2016-08-12

**Authors:** R. Shukla, S. J. Patwe, S. K. Deshpande, S. N. Achary, P. S. R. Krishna, A. B. Shinde, J. Gopalakrishnan, A. K. Tyagi

**Affiliations:** 1 Chemistry Division, Bhabha Atomic Research Centre, Mumbai-400085, India; 2 UGC-DAE Consortium for Scientific Research, Bhabha Atomic Research Centre, Mumbai-400085, India; 3 Solid State Physics Division, Bhabha Atomic Research Centre, Mumbai-400085, India; 4 Solid State and Structural Chemistry Unit, Indian Institute of Science, Bangalore-560 012 India

## Abstract

We report composition dependent structure evolution from SrTiO_3_ to SrFe_0.5_Ta_0.5_O_3_ by powder X-ray and neutron diffraction studies of SrTi_1−2x_Fe_x_Ta_x_O_3_ (0.00 ≤ × ≤ 0.50) compositions. Structural studies reveal cubic (Pm3m) perovskite-type structure of the parent SrTiO_3_ for x up to 0.075 and cation disordered orthorhombic (Pbnm) perovskite-type structure for x ≥ 0.33. A biphasic region consisting of a mixture of cubic and orthorhombic structures is found in the range for 0.10 ≤ × ≤ 0.25. Dielectric studies reveal transformation from a normal dielectric to relaxor like properties with increasing Fe^3+^ and Ta^5+^ concentration. Dielectric response is maximum at x = 0.33 in the series. The results establish a protocol for designing new lead-free relaxor materials based on the co-substitution of Fe^3+^ and Ta^5+^ for Ti^4+^ in SrTiO_3_. A complex interplay of strain effects arising from distribution of cations at the octahedral sites of the perovskite structure controls the dielectric properties.

The environment friendly lead free perovskite-type materials have been of attraction for several important properties, like ferroelectricity, high dielectric constant and magnetic properties. The observation of high dielectric permittivity over a wide temperature range in perovskite type materials stimulates research for discovery and processing of newer materials for technological applications^1^,^2^. Large dielectric permittivity (ε′ ~ 10^5^) has been reported for a cation ordered perovskite-type compound, namely CaCu_3_Ti_4_O_12_^3^. Later similar orders of permittivities have been reported in a number of perovskite type compounds with or without cation ordering^4^,^5^,^6^. Towards the aim for materials with larger dielectric constant, a number of studies on doped BaTiO_3_, KNbO_3_, Na_0.5_Bi_0.5_TiO_3_, A_2_FeBO_6_ (A = alkaline earth metal, B = Sb, Nb, Ta), etc. have been reported in literature^4^,^5^,^6^. Often it has been observed that the perovskite type materials with Ti, Nb and Ta exhibit high dielectric constant with near-zero temperature coefficients of resonant frequency and low dielectric loss^5^,^6^. Among the alkaline-earth perovskite type titanates, only SrTiO_3_ has an ideal primitive cubic structure at ambient temperature. The dielectric and structural properties of SrTiO_3_ have been investigated in several reports^4^,^5^,^6^,^7^,^8^. Though the SrTiO_3_ lattice is considered as a classical displacive system, the ferroelectric phase has not been observed at any temperature in this compound. Literature shows quantum ferroelectric behavior in SrTiO_3_ at low temperature which gets diffused by the substitution due to the formation of local polar clusters and lattice distortion induced by local strains^9^,^10^,^11^. Thus much research attention has been paid to create strained system either by doping or as thin films. Similar studies on other mixed cation perovskite-type solid solutions like CaAl_0.5_Nb_0.5_O_3_–CaTiO_3_ revealed that the dielectric properties vary drastically with compositions^12^. Furthermore, the non-magnetic (*d*^*0*^) cations in perovskite lattice can exhibit second order Jahn-Teller (SOJT) distortion which can lead to ferroelectric or relaxor-like properties^13^,^14^. The soft optical phonon modes introduced by such distortions under tensile and compressive stress are reflected as high static dielectric constant and ferroelectric properties in the incipient ferroelectric properties of TiO_2_ and SrTiO_3_^15^,^16^. Alternatively, creation of localized polar defects is also a known procedure adopted for design of materials where the localized carrier hopping lead to large static permittivity^17^,^18^. Larger permittivity in rutile-type TiO_2_ has also been achieved in lightly doping of In^3+^ and Nb^5+^ at Ti^4+^ site^19^. We had successfully demonstrated the transformation of the incipient ferroelectric, rutile (TiO_2_) to a relaxor ferroelectric by cosubstitution of Fe^3+^ and Ta^5+^ for Ti^4+^, while maintaining the charge compensation^20^,^21^,^22^. In our earlier study^20^, it is observed that the cation disorder and synergistic effects of d^0^ and d^n^ cations play a key role in the permittivity and relaxor like behavior of these materials. In a similar strategy to prepare new ferroelectric materials in an otherwise incipient ferroelectric SrTiO_3_, we explored SrTi_1−2x_Fe_x_Ta_x_O_3_ system and the results are presented in this manuscript.

## Results and Discussion

**Figure 1:**
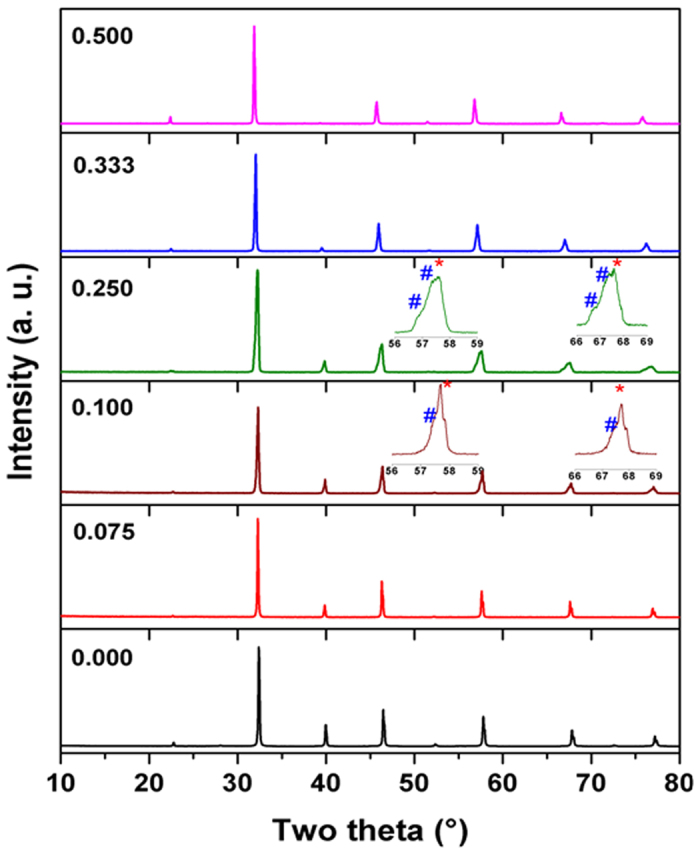
Powder XRD pattern of SrTi_1−2x_Fe_x_Ta_x_O_3_ compositions. (^#^ & * indicate orthorhombic and cubic phases, respectively).

The powder XRD patterns of representative compositions of SrTi_1−2x_Fe_x_Ta_x_O_3_ (0.0 ≤ × ≤ 0.5) series are shown in Fig. 1. The XRD data of the end members i.e. x = 0.0 and 0.5 could be assigned to the cubic SrTiO_3_ and orthorhombic SrFe_0.5_Ta_0.5_O_3_, respectively. As no distinct features attributable to cation ordering observed in the XRD data as well as neutron diffraction (ND) data explained later in this manuscript, we infer an orthorhombic cation disordered perovskite structure for x = 0.5 composition. The observed cation disordered structure for SrFe_0.5_Ta_0.5_O_3_ is in agreement with the earlier reports^23^,^24^,^25^. The powder XRD patterns recorded for the compositions with x = 0.0 to 0.075 are quite similar and they all could be explained by the primitive cubic SrTiO_3_ type structure. Similar analyses of the XRD patterns of the compositions with 0.33 ≤ × ≤ 0.5 revealed orthorhombic perovskite structures to all of them. However, the XRD patterns of the compositions with 0.10 ≤ × ≤ 0.25 shows asymmetrically broadened reflections with shoulder like features. With increasing x from 0.10, the intensities of shoulders observed at 2θ = 57.0 and 67.5°, increase systematically and they appeared as peaks attributable to orthorhombic phase.

As mentioned above, the two end members of the studied series have different structures, viz. cubic Pm3m at one end (x = 0.00) and orthorhombic Pbnm at other end (x = 0.5). These two structures are closely related and differ only by distortion and tilting of octahedral units. As the value of x increases, the structure is expected to transform from one structure to another either directly or through intermediate phases, like tetragonal, rhombohedral, monoclinic or triclinic etc. However, the observed XRD data of compositions with 0.1 ≤ × ≤ 0.25, suggest the absence of any such intermediate phases and could be better explained by considering a coexistence of the orthorhombic and cubic perovskite phases. Accordingly, the studied system could be grouped into three categories, namely, solid solution with cubic SrTiO_3_ type or orthorhombic SrFe_0.5_Ta_0.5_O_3_ type structures and mixture of these two solid solution phases. The unit cell parameters for the phases in the system, determined by refinement of the profile of powder XRD data, are given in Table 1.

In the single phase cubic and the orthorhombic phase regions a systematic increasing trend in unit cell volume with increasing Fe^3+^ and Ta^5+^ content is observed, which can be attributed to the larger ionic radii of Fe^3+^ and Ta^5+^ (rFe^3+^ = 0.645 Å and rTa^5+^ = 0.64 Å, in octahedral coordination) compared to Ti^4+^ ions (rTi^4+^ = 0.605 Å, in octahedral coordination). A varying trend of the observed unit cell parameters of both cubic and orthorhombic phases (Table 1) in biphasic region suggests that these phases did not reach the terminal solubility limit due to competition between these two closely related structures.

Further structural analyses of the compositions were carried out by Rietveld analyses of the powder ND data. The phase(s) and unit cell parameters observed from the XRD data were used to refine the ND data. Fifth order polynomial function and pseudo-Voigt profile function were used to model the background and Bragg peaks of the powder neutron diffraction data. The existence of three different phase fields is further confirmed from the powder ND data.

The refined unit cell parameters obtained from the ND data support the observation of the XRD data. In addition the analyses of the powder ND data considering distorted perovskite type structure of possible intermediate morphotropic phase boundary phases like Cm, Pm, R3m and P4mm^25^,^26^,^27^ were not successful. In general the electrical properties, like dielectric or relaxor ferroelectric properties are enhanced in such phase boundary compositions^26^. The observed dielectric properties explained later in the manuscript do not reveal any such enhanced dielectric constants. Thus, the absence of any single phase distorted structure in the phase boundary of cubic and orthorhombic compositions of studied samples is inferred.

**Figure 2:**
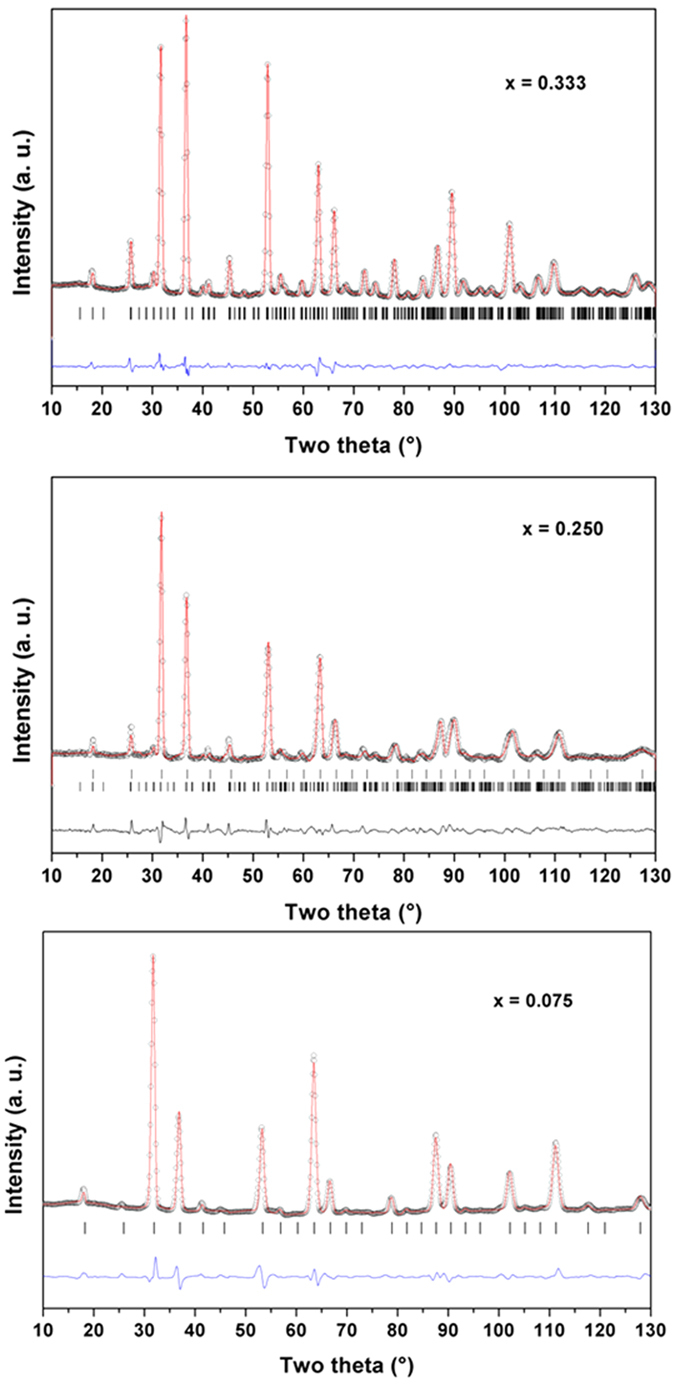
Rietveld refinement plots for powder ND data of representative compositions in SrTi_1−2x_Fe_x_Ta_x_O_3_ system. (x = 0.075, 0.25 and 0.33).

**Figure 3:**
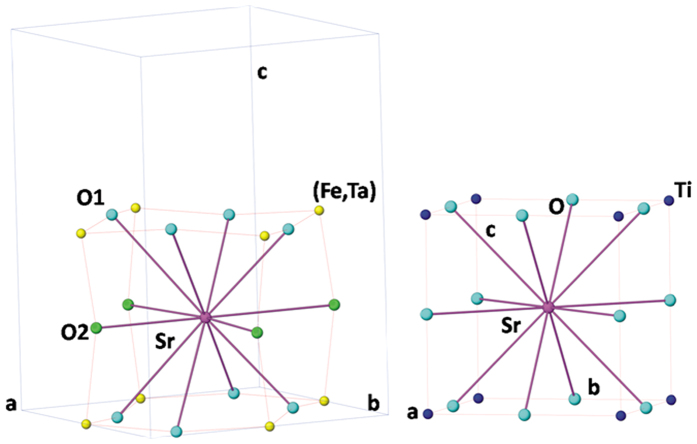
Representations for structures of orthorhombic (SrFe_0.5_Ta_0.5_O_3_) (left) and cubic (SrTiO_3_) (right).

The refinements of the occupancies of the single phase compositions do not show appreciable deviation from nominal stoichiometries. However, the refinement of occupancies of the phases in biphasic compositions, in particular the compositions with x = 0.10, 0.125 are not stable. Thus the nominal stoichiometries or stoichiometry of compositions close to the nearest single phase sample were considered for the refinements. Besides, a gradual increasing fraction of orthorhombic phase with increasing x is observed in the biphasic region. The structural parameters for different phases as obtained from the powder neutron diffraction data are summarized in the supplementary information Table S-1(a–c). Representative Rietveld refinement plots from ND data of the investigated system are shown in Fig. 2. Rietveld refinement fitted plots for other compositions are given in supplementary information (Figure S-I(a–j)). Typical inter-atomic distances are given as supporting information Table S2. As mentioned earlier the orthorhombic (*Pbnm*) and (*Pm3m*) cubic perovskite structures have closely similar atomic arrangements and the structural transition occurs by the cooperative tilting of the octahedral units (Fig. 3). The unit cell parameters of these two phases can be related as: a_o_ ~ b_o_ ~ a_p_ × √2; c_o_ ~ a_p_ × 2, where the subscript o and p represent orthorhombic and cubic structure, respectively. The analyses of structural parameters of the cubic solid solution phase revealed that all the cations are randomly distributed. A small increase in the M-O (M = Ti, Ta, Fe) and Sr-O bond lengths are observed with increasing x which can be accounted to the larger ionic radii of Fe^3+^ and Nb^5+^ compared to Ti^4+^. Similar analyses of the structural parameters of the orthorhombic phase compositions suggest that the distortion in octahedral units increases with the increasing Fe^3+^ and Ta^5+^ contents in the lattice. In addition, the inter-octahedral angles of the orthorhombic phases increase systematically with increasing the Fe^3+^ and Ta^5+^ in the compositions, viz. M-O1-M angles are: 171.5(1), 173.3(1) and 174.4(1)° for x = 0.333, 0.375 and 0.50, respectively; M-O2-M angles are: 164.34(6), 165.29(6) and 166.05(1)° for x = 0.333, 0.375 and 0.50 respectively). This trend of the inter-octahedral angles indicates a decrease in the tilt of MO_6_ octahedra with increasing values of x. This might be related to lager average ionic radii of Fe^3+^ and Ta^5+^ pair compared to that of Ti^4+^. Further, the larger differences in the ionic radii are also likely to cause local distortion in the octahedral units which would reflect in their electrical properties, in particular dielectric properties as discussed in subsequent section.

**Figure 4:**
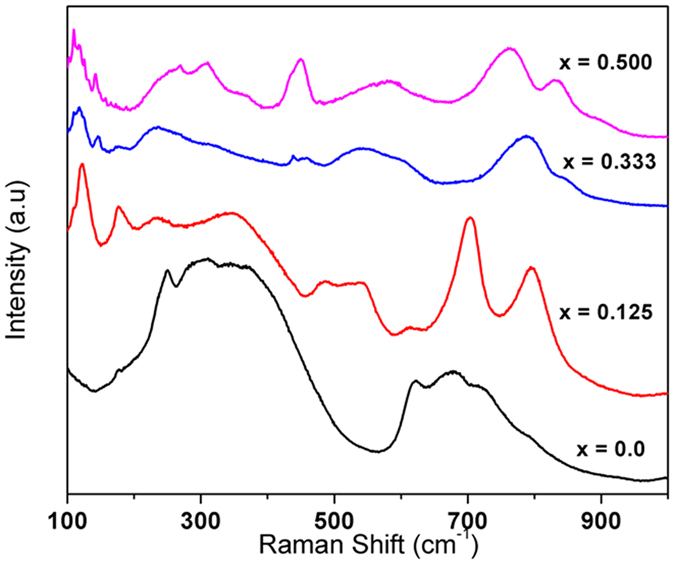
Raman spectra of SrTi_1−2x_Fe_x_Ta_x_O_3_ compositions (x = 0.0, 0.125, 0.33 and 0.50).

Further to support the evolution of structure with composition Raman spectroscopic studies were carried out on representative compositions, namely x = 0.00, 0.125, 0.333 and 0.50 and they are shown in Fig. 4. The composition with x = 0.00 composition (SrTiO_3_) should not show any Raman modes due to its cubic Pm3m (O1h) structure where all the atoms are occupied at the positions of inversion symmetry. However, the modes due to second-order scattering are usually observed as broad peaks centered at 300 and 650 cm^−1 ^28. The observed Raman spectrum of x = 0.00 is in agreement with such second order scattering process as reported in literature^28^. The orthorhombic (Pbnm) structure of x = 0.50 composition should exhibit 24 Raman active modes: 7A_g_ + 5B_1g_ + 7B_2g_ + 5B_3g_^29^. In the present Raman study, the modes observed at 109, 143, 256, 304, 445, 575, 764, 836 cm^−1^ can be attributed to orthorhombic perovskite type phase^29^. A comparison of the spectra of x = 0.333 and 0.50 indicates the closely similar features, which further support the orthorhombic structure for both the composition as inferred from the diffraction studies. The Raman spectrum of x = 0.125 composition shows different features compared to x = 0.333 and 0.50. The clear modes around 352, 479 and 703 cm^−1^ suggests distinct local distortion and bonding around the cations compared to orthorhombic and cubic phases. The redistribution of octahedral cations in the cubic and orthorhombic phase might also be origin of such differences.

In our earlier studies, it has been reported that the Fe containing rutile-type titanates compositions often show different electrical properties compared to the analogous Cr and Ga containing compositions^20^,^21^,^22^. The possible existence of heterovalent Fe might be a reason for such differences. In the Fe and Ti containing compositions, elctronic defects can be created due to the reduction of Fe^3+^ to Fe^2+^ as well as Ti^4+^ to Ti^3+^. However due to the differences in the reduction potential of Fe^3+^-Fe^2+^ and Ti^4+^-Ti^3+^ redox couple, the reduction is more probable for Fe^3+^ compared to Ti^4+^. Alternatively, the structure may retain subtle amounts of oxygen vacancies due to such reduction which is not detected by neutron diffraction. The localized holes or oxygen vacancies may form disordered short range polar domains and control their electrical properties. Additional interfacial polarizations from the grain boundaries of the biphasic systems are also likely to affect the dielectric properties as explained in subsequent section.

**Figure 5:**
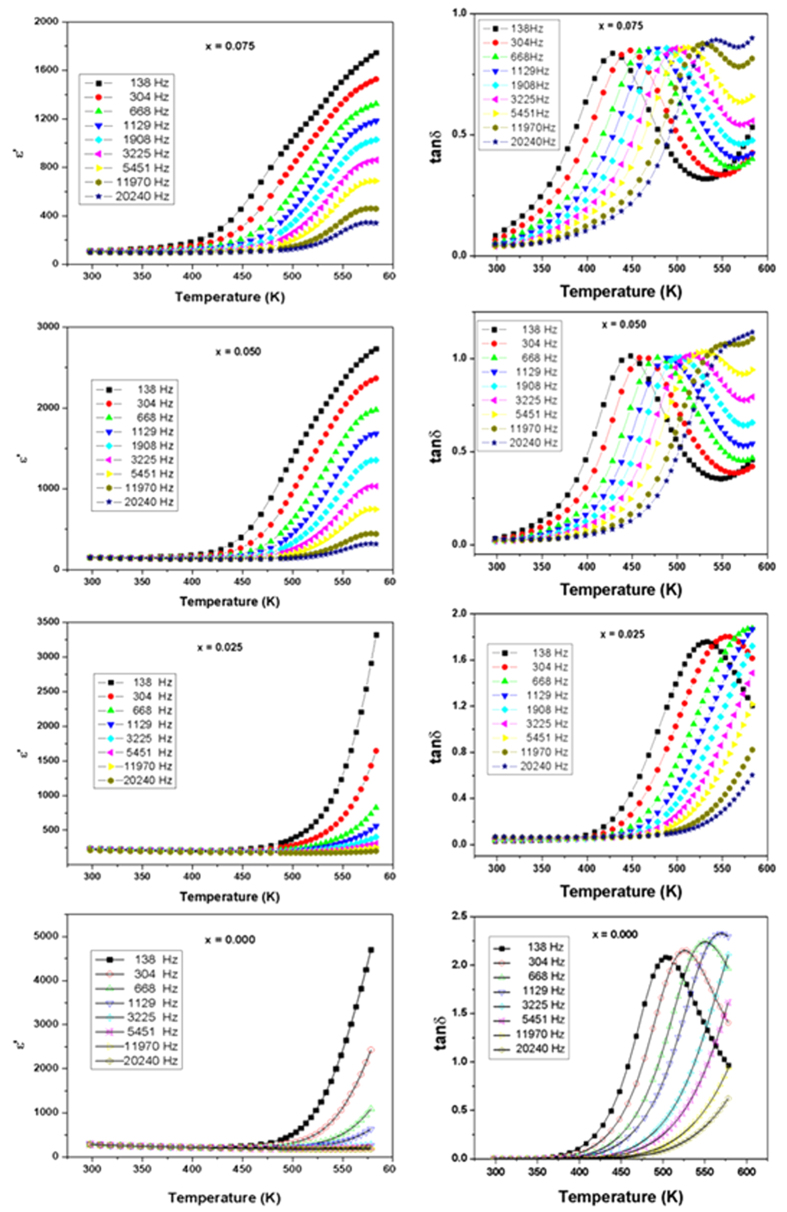
Variation of real part of relative permittivity and tan δ with temperature for cubic solid solution phases in SrTi_1−2x_Fe_x_Ta_x_O_3_ compositions (x = 0.0, 0.025, 0.050 and 0.075). The solid lines are guide to the eye.

The three different phase regions observed in the SrTi_1−2x_Fe_x_Ta_x_O_3_ (0 ≤ × ≤ 0.5) compositions show three distinct types of dielectric responses and ferroelectric properties. The temperature dependent real part of dielectric permittivity (ε′) and loss tangent (tan δ) of the single phase cubic solid solution compositions, viz. for 0.00 ≤ × ≤ 0.075, at several frequencies are shown in Fig. 5.

The normal dielectric like behavior for SrTiO_3_ can be inferred from the monotonically increasing trend of ε′ with the increase in temperature. The high temperature permittivities systematically decrease with the increase in the values of x in cubic solid solution phases. The rapidly increasing trend of ε′ at higher temperature for x = 0.00 composition is not observed in the co-substituted compositions.

At higher frequency, the composition with x = 0.050 and 0.075 show peak like features at higher temperature (~550 K) which is similar to a ferroelectric phase transition. However, the broad nature of the peak and shifting of the position of peak maximum with frequency as well as absence of peak or more close to a step like feature at lower frequency, suggest no long range dipolar ordering but rather implies local polar unit or defects. The polarization versus electric field measurements on these compositions show ferroelectric like loop opening but the loops do not appear to saturate (Supplementary Information Figure S-II). The shape and features of these PE loops further confirms absence of classical ferroelectricity.

**Figure 6:**
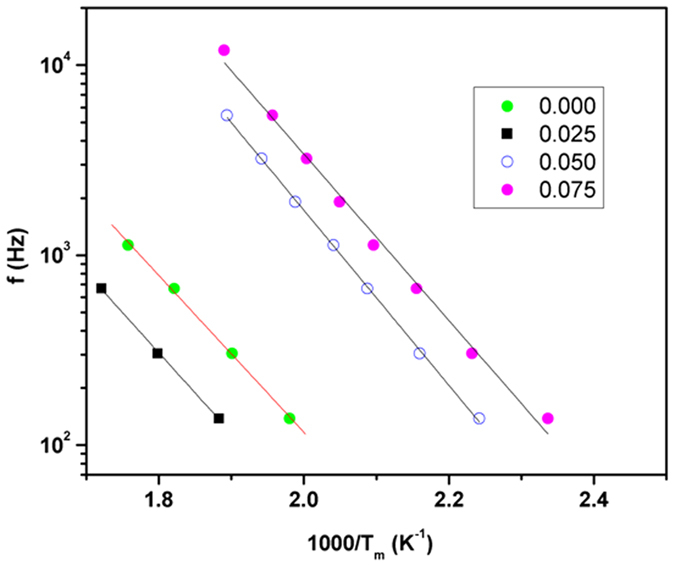
Variation of peak maxima of relaxation peak (T_m_) with frequency for cubic SrTi_1−2x_Fe_x_Ta_x_O_3_ compositions (x = 0.0, 0.025, 0.050 and 0.075) compositions. Solid lines are fits to the Arrhenius relation.

Despite the similarities in the structures, the systematic decrease in dielectric permittivity at higher temperature suggests an appreciable contribution of defects, like anion vacancies towards the dielectric properties. Further to understand the decreasing permittivity the loss tangents of the compositions have been analyzed. In all these compositions, the relaxation peaks in the temperature dependent *tan δ* shift to higher temperature with the increasing frequency. Also the magnitude of the peak increases with the increasing frequency which indicates that the loss is due to the hopping conduction and not due to Maxwell-Wagner interfacial polarization, as the latter effect is larger at lower frequencies compared to that at higher frequencies. The variation of T_m_ (temperature corresponding to peak maximum in the variation of tan δ with temperature) with frequency for these compositions shows a typical Arrhenius type (eqn-1) behavior and they are depicted in Fig. 6.


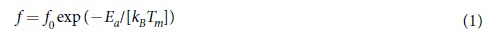


where *f*_*0*_ is a pre-exponential factor, *k*_*B*_ is the Boltzmann constant, *T*_*m*_ is the temperature at *tan δ* peaks and *E*_*a*_ is the activation energy.

The typical activation energies for relaxation process observed in the cubic single phase compositions, i.e. for x = 0.00, 0.025, 0.050 and 0.075 are: 0.82, 0.84, 0.91 and 0.87 eV, respectively. The larger values of activation energies suggest ionic conduction and possibly hopping of oxide ions is the origin of the relaxation.

**Figure 7:**
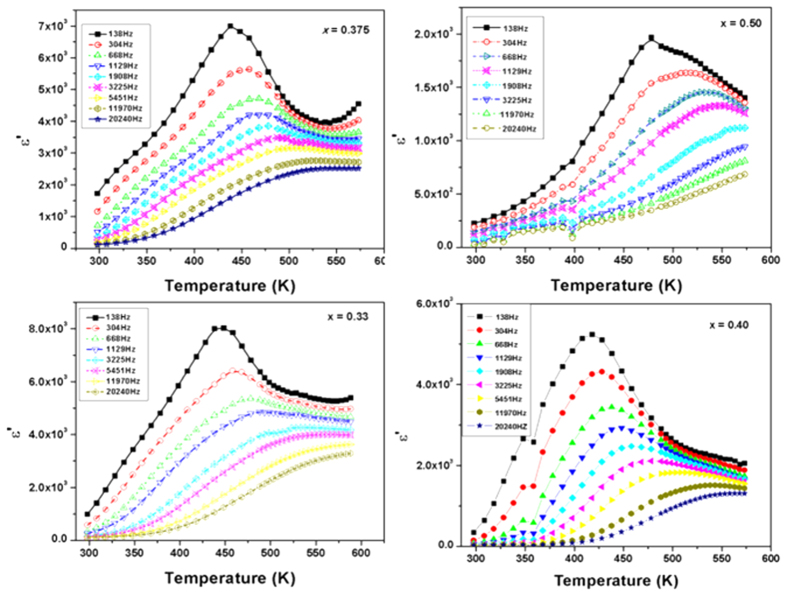
Variation of real part of relative permittivity with temperature for orthorhombic solid solution phases in SrTi_1−2x_Fe_x_Ta_x_O_3_ system (x = 0.33, 0.375, 0.40 and 0.50). Solid lines are guide to the eye.

Frequency and temperature dependent dielectric studies on the orthorhombic single phase compositions, i.e. with 0.33 ≤ × ≤ 0.50 show a very different behavior. At selected frequencies, the variations of real part of the relative permittivity (ε′) with temperature are shown in Fig. 7. Broad peaks with strong frequency dependence are observed for these compositions. The peak permittivity increases significantly with the increase in Ti content, e.g. for x = 0.333, 0.375, 0.40, and 0.50, the values of ε′ are about 8000, 7000, 5225, and 2000, respectively (at 138 Hz). Interestingly, the dispersion of ε′ decreases significantly and the curves come closer at temperatures *T* higher than *T*_*m*_ (temperature at maximum ε′).

**Figure 8:**
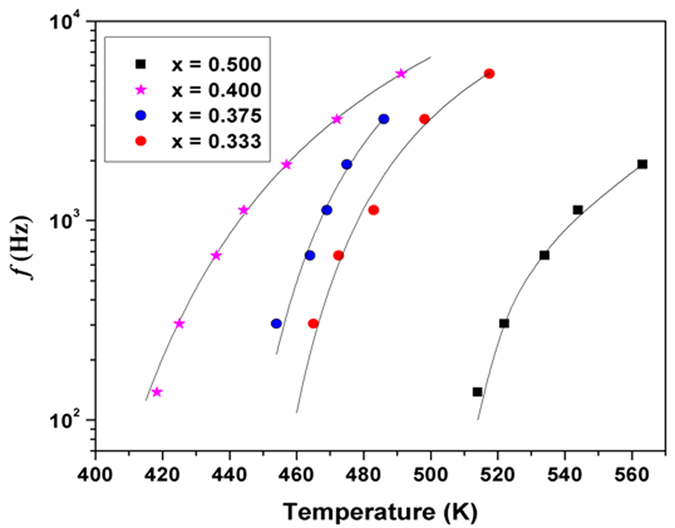
Variation of peak maxima of relaxation peak (T_m_) with frequency for orthorhombic solid solution phases in SrTi_1−2x_Fe_x_Ta_x_O_3_ system. Solid lines are fits to the Vogel-Fulcher relation.

The strong dispersion below T_m_ and merging of the curves above T_m_ resembles the characteristic features of a diffuse phase transition (DPT) of relaxor ferroelectrics^20^,^21^,^22^,^26^. Attempts to fit the relaxation frequency and *T*_*m*_ with Arrhenius relation (eqn-1) were not successful, but they could be related by Vogel-Fulcher (V-F) relation (eqn-2). The result of fitting of eqn. 2 to the frequency-temperature behavior is shown in Fig. 8. The Vogel-Fulcher type behavior of the frequency dependency of the T_m_ confirms the relaxor nature of the orthorhombic phase compositions.


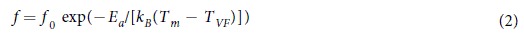


where *f*_*0*_ is a pre-exponential factor, *k*_*B*_ is the Boltzmann constant, *T*_*VF*_ is the Vogel-Fulcher temperature and *E* is the activation energy.

The best-fit values obtained for sample with x = 0.50 were *f*_*0*_ = 2.75 × 10^4^ Hz, *E* = 0.024 eV and *T*_*VF*_ = 457 K. Corresponding values for the other samples are as: *f*_*0*_ = 1.54 × 10^5^ Hz, *E* = 0.040 eV and *T*_*VF*_ = 348 K (for x = 0.40); *f*_*0*_ = 6.5 × 10^4^ Hz, *E* = 0.020 eV and *T*_*VF*_ = 418 K (for x = 0.375); *f*_*0*_ = 4.8 × 10^4^ Hz, *E* = 0.017 eV and *T*_*VF*_ = 428 K (for x = 0.33). These observations strongly support the classical relaxor nature of these samples. The observed pre-exponential factor (*f*_*0*_), i.e. the attempt frequency observed in V-F fitting have smaller values compared to that observed for relaxor ferroelectrics (usually in the range of 10^10^ Hz).

The values of attempt frequency indicate size and interaction of polar domains in the dielectric material. For a relaxor ferroelectric the larger domain size and stronger interaction usually show lower value of attempt frequency. In general, the relaxation by polaron hopping show higher value of attempt frequency, while the relaxation due to ionic hopping show lower attempt frequency, viz. 10^2^–10^3 ^30. In these studied compositions, the long range ionic conduction along with polaron conduction is expected and thus they may show lower relaxation frequency and also higher loss.

**Figure 9:**
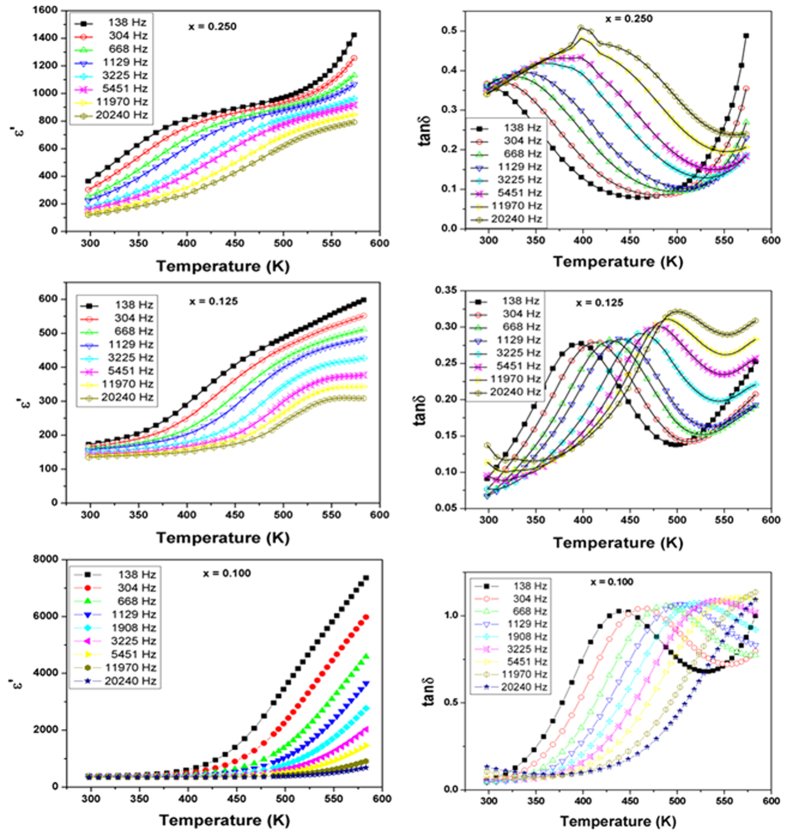
Variation of real part of relative permittivity and tan δ with temperature for biphasic compositions in SrTi_1−2x_Fe_x_Ta_x_O_3_ compositions (x = 0.10, 0.125, and 0.25).

**Figure 10:**
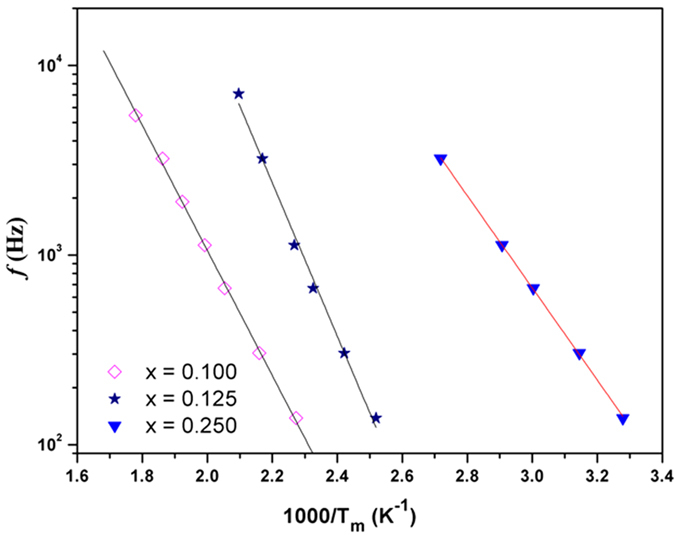
Variation of peak maxima of relaxation peak (T_m_) with frequency for biphasic compositions in SrTi_1−2x_Fe_x_Ta_x_O_3_ compositions (x = 0.10, 0.125, and 0.25). Solid lines are fit to the Arrhenius relation.

The frequency and temperature dependent dielectric studies on the biphasic compositions, i.e. with 0.100 ≤ × ≤ 0.25 revealed a different behavior compared to the compositions with single phase cubic and orthorhombic structures. The temperature dependence of ε′ of the mixed phase compositions are shown in Fig. 9. At ambient temperature, the values of the dielectric constant in these compositions are about 200 to 400. A rapid increase in permittivity at higher temperature suggests an onset of predominant conductivity contribution. A step like discontinuity is observed in the variation ε′ with temperature for the nominal composition with x = 0.25, while the same is gradually smeared and shifted towards higher temperature with the decreasing x. Also, in each of these compositions the position of step shifts towards higher temperature with increasing frequency. The relaxation features are clearly observed in the variation of loss tangents with temperature (Fig. 9). The magnitudes of the relaxation peaks observed in the tan *δ* vs T plots show a slight increasing trend with increasing frequency. This suggests that the relaxation corresponding to the step may be due to either hopping conduction or Maxwell-Wagner interfacial polarization or both. The Maxwell-Wagner interfacial polarization due to the interfaces of the two coexisting phases can be expected for these biphasic systems. However, the lower values of ε′ as well as *tan δ* at lower frequencies suggest no appreciable contributions from the Maxwell-Wagner interfacial polarization. The variations of peak positions in the tan δ vs. temperature plots with frequency are shown in Fig. 10, which show typical Arrhenius type behavior (eqn 1) similar to the cubic single phase compositions. The activation energies for the compositions with x = 0.10, 0.125 and 0.25 are: 0.66, 0.80 and 0.48 eV, respectively. These values are again similar to those expected for hopping conductivity of ions.

A comparison of the dielectric behavior of the studied compositions indicates that the Ti rich compositions show ferroelectric like behavior due to local distortion or strain while the Fe and Ta rich compositions show normal relaxor like behavior. Moreover, the relaxor response of the Fe and Ta containing compositions increases significantly with the increase in cation disorder. Maximum dielectric permittivity is observed for larger cation disordered single phasic orthorhombic composition, i.e. with x = 0.333. Thus, it can be suggested that the cation-disorder in the octahedral sites have a dominant role on the dielectric properties. Further the differences in the ionic radii of the cations in octahedral sites lead to different distortion in different structure types and in turn responses differently to the *ac* frequency. The ferroelectric like behavior of Ti rich compositions might be due to the local distortion induced by internal pressure arising from the larger cations, like Fe^3+^ and Ta^5+^. The activation energies of the relaxation process suggest that the relaxation originates from the hopping conductions of oxide ions. In the biphasic compositions, the grain boundaries of the coexisting phase might be assisting the conduction process and hence likely to result lower activation energy for relaxation. In the orthorhombic solid solution region, the relaxor like dielectric properties and polaron conduction is observed, which is closely similar to our earlier results on rutile based relaxor dielectrics^20^,^21^,^22^. Further it can be inferred that cation disorder and a significant concentration of Fe are desired parameters for relaxor like properties and their optimum concentration can lead to a higher permittivity. These findings may be useful in designing new lead-free relaxor ferroelectric materials.

## Methods Section

Nominal compositions with stoichiometry SrTi_1−2x_Fe_x_Ta_x_O_3_ (0.0 ≤ × ≤ 0.5) were prepared by solid state reaction of appropriate amounts of SrCO_3_, Fe_2_O_3_, Ta_2_O_5_ and TiO_2_. Homogenous mixtures of reactants were heated at 1173 K for 30 h in static air followed by regrinding and heating at 1473 K for 30 h in pellet form. The products were reground and pressed into pellets (10 mm diameter and 2 mm thickness) and sintered at 1623 K for 30 h. The final sintered products were characterized by powder X-ray diffraction (XRD) and neutron diffraction (ND) methods. The powder XRD patterns were recorded in the range of two theta 10–80° on a powder X-ray diffractometer (Panalytical, Model: X-pert pro) using CuKα radiation. The step width and counting time for the XRD data collections were 0.02° and 4 sec/step. Silicon was used as an external standard for calibration of diffractometer. Powder Neutron Diffraction data were collected on a 5 linear PSD based Debye-Scherrer type diffractometer installed at Dhruva research reactor, Bhabha Atomic Research Centre, Mumbai. The diffraction data were collected in the two range of 5 to 135° using neutrons of wavelength 1.2443 Å in a time span of 24 h. The recorded XRD and ND data of the compositions were analyzed by Rietveld refinement methods using Fullprof-2k software package. Raman spectrum of polycrystalline sample was excited using 532 nm line. Backscattered light was analyzed using a home built 0.9 m single monochromator, coupled with an edge filter and detected by a cooled CCD. Entrance slit was kept at 50 μm, which gives a spectral band pass of 3 cm^−1^. Dielectric measurements were carried out on the samples in cylindrical pellet form using Novocontrol Alpha impedance analyzer (Novocontrol Technologies, Germany) equipped with a Quatro liquid nitrogen gas cryosystem. The dielectric measurements were performed over a frequency range of 100 Hz to 5 MHz at several temperatures while heating from room temperature. Silver paint was applied to the flat surfaces of samples for proper electrical contact, and the pellets were mounted between flat gold-plated electrodes in a parallel-plate.

## Additional Information

**How to cite this article**: Shukla, R. *et al*. Structural manipulation and tailoring of dielectric properties in SrTi_1−x_Fe_x_Ta_x_O_3_ perovskites: Design of new lead free relaxors. *Sci. Rep.* **6**, 23400; doi: 10.1038/srep23400 (2016).

## Supplementary Material

Supplementary Information

## Figures and Tables

**Table 1:** Phases and refined unit cell parameters (from powder XRD data) of SrTi_1−2x_Fe_x_Ta_x_O_3_ (0.00 ≤ × ≤ 0.50) compositions.

x	Cubic (Pm3m)		Orthorhombic (Pbnm)			
	a (Å)	V (Å)^3^	a (Å)	b (Å)	c (Å)	V (Å)^3^
0.000	3.9052(1)	59.557(1)	**--------**	**--------**	**--------**	**--------**
0.025	3.9083(1)	59.700(3)	**--------**	**--------**	**--------**	**--------**
0.050	3.9121(1)	59.874(3)	**--------**	**--------**	**--------**	**--------**
0.075	3.9159(1)	60.046(2)	**--------**	**--------**	**--------**	**--------**
0.100	3.9105(3)	59.799(3)	5.5666(5)	5.5429(7)	7.8350(11)	241.75(5)
0.125	3.9145(1)	59.984(1)	5.5944(7)	5.5361(6)	7.8300(6)	242.50(4)
0.250	3.9257(2)	60.498(4)	5.6070(5)	5.5825(8)	7.8672(11)	246.25(5)
0.333	--------	--------	5.6061(2)	5.5799(2)	7.8894(2)	246.79(1)
0.375	--------	--------	5.6090(2)	5.5877(2)	7.8994(2)	247.58(1)
0.400	--------	--------	5.6034(1)	5.5963(2)	7.9135(2)	248.16(1)
0.450	--------	--------	5.6172(1)	5.6068(1)	7.9135(2)	249.45(1)
0.500	--------	--------	5.6235(2)	5.6118(1)	7.9268(2)	250.15(1)
