# Supplementary material for: Structural manipulation and tailoring of dielectric properties in SrTi1−xFexTaxO3 perovskites: Design of new lead free relaxors
Source: Sci Rep. 2016 Aug 12;6:23400. doi: 10.1038/srep23400 (PMC4981879; doi:10.1038/srep23400)
Supplement: Supplementary Information [file srep23400-s1.pdf]

Supplementary Information

Structural manipulation and tailoring of dielectric properties in $SrTi_{1-x}Fe_xTa_xO_3$ perovskites: Design of new lead free relaxors

R. Shukla[1], S. J. Patwe[1], S. K. Deshpande[2], S. N. Achary[1], P. S. R. Krishna[3], A. B. Shinde[3], J. Gopalakrishnan[4] and A. K. Tyagi[1]*

[1]Chemistry Division, Bhabha Atomic Research Centre, Mumbai-400085, India

[2]UGC-DAE Consortium for Scientific Research, Bhabha Atomic Research Centre, Mumbai-400085, India

[3]Solid State Physics Division, Bhabha Atomic Research Centre, Mumbai-400085, India

[4]Solid State and Structural Chemistry Unit, Indian Institute of Science, Bangalore-560 012 India

Supplementary Tables

Table S1: Refined structural parameters of selected $SrTi_{1-2x}Fe_xTa_xO_3$ ($0.00 \le x \le 0.50$) compositions. (PND: Powder Neutron diffraction, XRD: powder XRD data)

(a). for $0.00 \le x \le 0.075$ (Cubic solid solution); C: Cubic (Pm3m); Sr: *(0,0,0);* Ti: *(½,½,½);*O1: *(½,½,0)*

(b). for $0.333 \le x \le 0.5$ (Orthorhombic solid solution); O: Orthorhombic (Pbnm) Sr: *(x,y,¼);* M: *(½,0, ½);* O1: *(x,y,z);* O2*: (x,y,¼)*

(c). for $0.1 \le x \le 0.25$ (Coexisting Cubic and Orthorhombic solid solution)

Table S2: Typical inter-atomic distances (Å) and angles (^{o}) in $SrTi_{1-2x}Fe_xTa_xO_3$ compositions.

Supplementary Figures

Fig. S-I(a-j). Rietveld refinement plot of powder neutron diffraction data for $SrTi_{1-2x}Fe_xTa_xO_3$

(a). Rietveld refinement plot for x = 0.00 ($SrTi_{1.00}Fe_{0.00}Ta_{0.00}O_3$). Cubic (Pm3m)

(b). Rietveld refinement plot for x = 0.05 ($SrTi_{0.95}Fe_{0.025}Ta_{0.025}O_3$). Cubic (Pm3m) (vertical ticks for Bragg position)

(c).Rietveld refinement plot for x = 0.05 ($SrTi_{0.90}Fe_{0.05}Ta_{0.05}O_3$). Cubic (Pm3m) (vertical ticks for Bragg position).

(d).Rietveld refinement plot for x = 0.075 ($SrTi_{0.85}Fe_{0.075}Ta_{0.075}O_3$). Cubic (Pm3m) (vertical ticks for Bragg position)

(e).Rietveld refinement plot for x = 0.10 ($SrTi_{0.80}Fe_{0.10}Ta_{0.10}O_3$) (vertical ticks for Bragg position: Cubic (upper) and Orthorhombic (lower))

(f).Rietveld refinement plot for x = 0.125 ($SrTi_{0.75}Fe_{0.125}Ta_{0.125}O_3$), (vertical ticks for Bragg position: Cubic (upper) and Orthorhombic (lower))

(g).Rietveld refinement plot for x = 0.25 ($SrTi_{0.50}Fe_{0.25}Ta_{0.25}O_3$), (vertical ticks for Bragg position: Cubic (upper) and Orthorhombic (lower))

(h).Rietveld refinement plot for x = 0.333 ($SrTi_{0.333}Fe_{0.333}Ta_{0.333}O_3$): Orthorhombic (Pbnm) (vertical ticks for Bragg position)

(i).Rietveld refinement plot for x = 0.375 ($SrTi_{0.25}Fe_{0.375}Ta_{0.375}O_3$): Orthorhombic (Pbnm) (vertical ticks for Bragg position)

(j).Rietveld refinement plot for x = 0.50 ($SrTi_{0.00}Fe_{0.50}Ta_{0.50}O_3$): Orthorhombic (Pbnm) (vertical ticks for Bragg position)

Figure S-II: Polarization vs Electric field loops of cubic solid solution phases of $SrTi_{1-2x}Fe_xTa_xO_3$ ($0.00 \le x \le 0.075$) compositions. Measured at the frequency 150 Hz and at ambient temperature by using Aixacct TF2000 (Aixacct GmbH, Germany) ferroelectric analyzer.

Table S1: Refined structural parameters of selected $SrTi_{1-2x}Fe_xTa_xO_3$ ($0.00 \leq x \leq 0.50$) compositions. (PND: Powder Neutron diffraction, XRD: powder XRD data)

(a). for $0.00 \leq x \leq 0.075$ (Cubic solid solution); : C: Cubic (Pm3m); Sr: *(0,0,0);* Ti: *(½,½,½);*O1: *(½,½,0)*

x =	0.000	0.025	0.050	0.075
Data	PND	PND	PND	PND
Phase	C	C	C	C
Str.	Pm3m	Pm3m	Pm3m	Pm3m
Sr				
Occ.	1	1	1	1
B $(Å)^2$	0.50(2)	0.74(3)	0.77(3)	0.53(4)
Ti/Fe/Ta				
Occ (Ti:Fe:Ta)	1.00:0.00:0.00	0.95:0.025:0.025	0.90:0.05:0.05	0.85:0.075:0.075
B $(Å)^2$	0.19(4)	0.25(5)	0.11(5)	0.16(6)
O1				
Occ.	1	1	1	1
B$(Å)^2$	0.61(2)	0.74(2)	0.85(2)	0.98(2)
a (Å)	3.9049(1)	3.9078(1)	3.9116(1)	3.9161(1)
V $(Å)^3$		59.670(3)	59.850(3)	60.055(4)
Rp	3.63	3.49	2.71	3.28
Rwp	5.18	5.12	3.92	5.02
Rexp	3.03	1.81	1.81	1.81
χ^2	2.92	7.97	4.66	7.66
R_B	5.58	6.92	3.26	2.68

(b). for 0.333 ≤ x ≤ 0.5 (Orthorhombic solid solution);, O: Orthorhombic (Pbnm) Sr *(x,y,¼);* M: *(½,0, ½);* O1: *(x,y,z);* O2 *: (x,y,¼)*

x =	0.333	0.375	0.400	0.450	0.500
Data	PND	PND	XRD	XRD	PND
Phase	O	O	O	O	O
Str.	Pbnm	Pbnm	Pbnm	Pbnm	Pbnm
Sr *(x,y,1/4)*	0.0069(5)	0.0080(5)	-0.0003(8)	0.0009(6)	0.0053(5)
	0.0093(5)	0.0103(5)	0.0036(6)	0.0072(3)	0.0129(5)
	0.25	0.25	0.25	0.25	0.25
Occ.	1	1		1	1
B (Å)^2	0.84(2)	0.79(2)		-	0.83(2)
Ti/Fe/Ta *(1/2,0,1/2)*	0.5	0.5	0.5	0.5	0.5
	0	0	0	0	0
	0.5	0.5	0.5	0.5	0.5
Occ (Ti:Fe:Ta)	0.33:0.33:0.33	0.25:0.375:0.375	0.20:0.40:0.40	0.10:0.45:0.45	0.0:0.5:0.5
B (Å)^2	0.76(3)	0.66(3)		-	0.59(2)
O1	0.2435(4)	0.2421(4)	0.2418(8)	0.2419(8)	0.2386(4)
	0.2455(4)	0.2446(4)	0.2442(8)	0.2443(8)	0.2417(4)
	0.0109(3)	0.0120(3)	0.0152(6)	0.0145(6)	0.0159(3)
Occ.	1	1	1	1	1
B(Å)^2	0.65(2)	0.67(3)	-	-	0.63(4)
O2	0.9573(4)	0.9519(5)	0.9509(9)	0.9529(8)	0.9525(5)
	0.5054(6)	0.5067(6)	0.5003(17)	0.5040(14)	0.5099(6)
	0.25	0.25	0.25	0.25	0.25
Occ.	1	1		1	1
B(Å)^2	1.53(7)	1.25(8)		-	1.3(1)
			B_{ov}: 0.42(1) (Å)^2	B_{ov}: 0.42(1) (Å)^2	
a (Å)	5.605(1)	5.611(1)	5.6038(1)	5.6171(1)	5.619(1)
b (Å)	5.580(1)	5.585(1)	5.5963(1)	5.6068(1)	5.616(1)
c (Å)	7.888(1)	7.898(2)	7.9132(1)	7.9204(1)	7.928(2)
V (Å)^3	246.7(1)	247.5(1)	248.17(1)	249.45(1)	250.2(1)
R_p, R_{wp}, R_{exp}, χ^2	1.64, 2.36, 2.03, 1.36	2.63, 3.40, 1.92, 3.14	6.23, 8.21,10.09, 3.66	6.12, 8.02, 9.90, 2.60	2.72, 3.68, 2.02, 3.32
R_B	4.01	6.13	2.23	3.17	5.13

(c). for $0.1 \le x \le 0.25$ (Coexisting Cubic and Orthorhombic solid solution)

x =	0.1		0.125		0.25	
Data	PND		PND		PND	
Phase	C	O	C	O	C	O
Str.	Pm3m	Pbnm*	Pm3m	Pbnm	Pm3m	Pbnm
Wt.(%)	98(1) %	2(1)%	53(1)	47(1)	22(1)	78
Sr (x,y,z)	0	0.0087	0	0.0087(8)	0	0.0038
	0	0.0090	0	0.0090(8)	0	0.0061
	0	0	0	0	0	0
Occ.	1	1	1	1	1	1
B (Å)^2	0.50(4)	0.6	0.55(5)	0.6(2)	0.84(5)	1.36(2)
Ti/Fe/Ta	0.5	0.5	0.5	0.5	0.5	0.5
	0.5	0	0.5	0	0.5	0.5
	0.5	0.5	0.5	0.5	0.5	0.5
Occ (Ti:Fe:Ta)	0.80:0.10:0.10:	0.66:0.17:0.17	0.80:0.10:0.10	0.66(2):0.17(2):0.17(2)	0.80:0.10:0.10	0.46(2):0.22(2):0.22(2)
B (Å)^2	0.12(5)	0.58	0.13(6)	0.58(2)	0.20(6)	0.44(3)
O1	0.5	0.2416	0.5	0.2416(7)	0.5	0.2436(5)
	0.5	0.2437	0.5	0.2437(7)	0.5	0.2453(5)
	0	0.0066	0	0.0066(6)	0	0.0091(7)
Occ.	1	1	1	1	1	1
B(Å)^2	1.03(2)	0.4)	0.71(7)	0.4(1)	0.64(5)	0.94(3)
O2		0.9538		0.9538(9)		0.9600(5)
		0.5039		0.5039(10)		0.5005(7)
		0.25		0.25		0.25
Occ.		1		1		1
B(Å)^2		2.6		2.6(2)		1.96(4)
a (Å)	3.9096(2)	5.5666	3.9152(2)	5.597(2)	3.9257(1)	5.6070(2)
b (Å)	3.9096(2)	5.5429	3.9152(2)	5.543(2)	3.9257(1)	5.5825(2)
c (Å)	3.9096(2)	7.8350	3.9152(2)	7.848(4)	3.9257(1)	7.8672(4)
V (Å)^3	59.756(4)	241.747	60.02(1)	243.5(2)	60.498(1)	246.2(5)
R_p, R_{wp}, χ^2	3.58, 5.13, 7.98		2.32, 3.30, 2.48		3.98, 5.18, 7.17	
R_B	3.49	18.4	1.83	9.02	5.87	9.35

* Structural parameters observed at x = 0.125 were used.

Table S2: Typical inter-atomic distances (Å) and angles (^{o}) in $SrTi_{1-2x}Fe_xTa_xO_3$ compositions.

x =	0.000	0.025	0.050	0.075	0.100	0.1250		0.25		0.333	0.375	0.500	
Str.	Pm3m	Pm3m	Pm3m	Pm3m	Pm3m	Pm3m	Pbnm	Pm3m	Pbnm	Pbnm	Pbnm	Pbnm	
M-O1 × 6	1.952	1.955	1.957	1.956	1.957	1.958	1.962	1.963	1.973	1.979(2)	1.972(2)	1.972(2)	M-O1 × 2
							1.980		1.987	2.004(2)	1.992(2)	1.987(2)	M-O1 × 2
							1.962		1.980	2.001(1)	1.993(1)	1.987(3)	M-O2 × 2
							1.974		1.980	1.995(1)	1.986(1)	1.982(1)	$\langle M\text{-}O\rangle_6$
							0.17×10^{-4}		0.088×10^{-4}	0.299×10^{-4}	0.241×10^{-4}	0.129×10^{-4}	Distortion
Sr-O1 × 12	2.761	2.764	2.767	2.770	2.768	2.769	2.654	2.776	2.681	2.610(3)	2.640(3)	2.656(3)	Sr-O1 × 2
							2.858		2.778	2.931(3)	2.895(3)	2.868(3)	Sr-O1 × 2
							2.787		2.865	2.799(3)	2.775(3)	2.772(3)	Sr-O1 × 2
							2.824		2.837	2.892(3)	2.877(3)	2.879(3)	Sr-O1 × 2
							2.817		2.833	2.840(4)	2.830(4)	2.826(4)	Sr-O2
							2.760		2.771	2.807(4)	2.790(4)	2.782(4)	Sr-O2
							2.589		2.601	2.572(4)	2.581(4)	2.602(4)	Sr-O2
							3.009		3.007	3.047(4)	3.031(4)	3.004(4)	Sr-O2
							2.785		2.794	2.811(1)	2.801(1)	2.797(1)	$\langle Sr\text{-}O\rangle_{12}$
							14.84×10^{-4}		13.31×10^{-4}	24.89×10^{-4}	19.61×10^{-4}	15.60×10^{-4}	Distortion:
Ti-O1-Ti	180	180	180	180	180					171.45(10)	173.73(10)	174.41(9)	M-O1-M
										164.34(6)	165.29(6)	166.05(12)	M-O2-M

Supplementary Figures

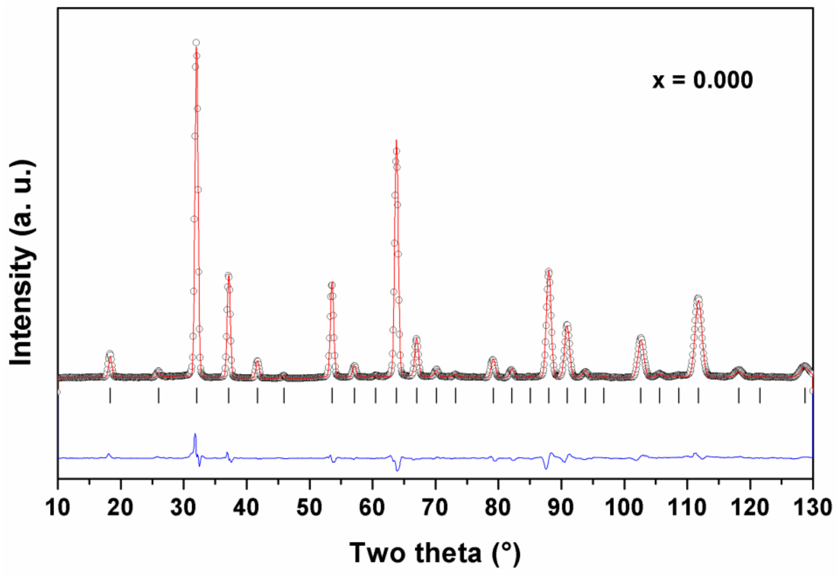

Fig. S-I(a). Rietveld refinement plot for x = 0.00 ($SrTi_{1.00}Fe_{0.00}Ta_{0.00}O_3$). Cubic (Pm3m)

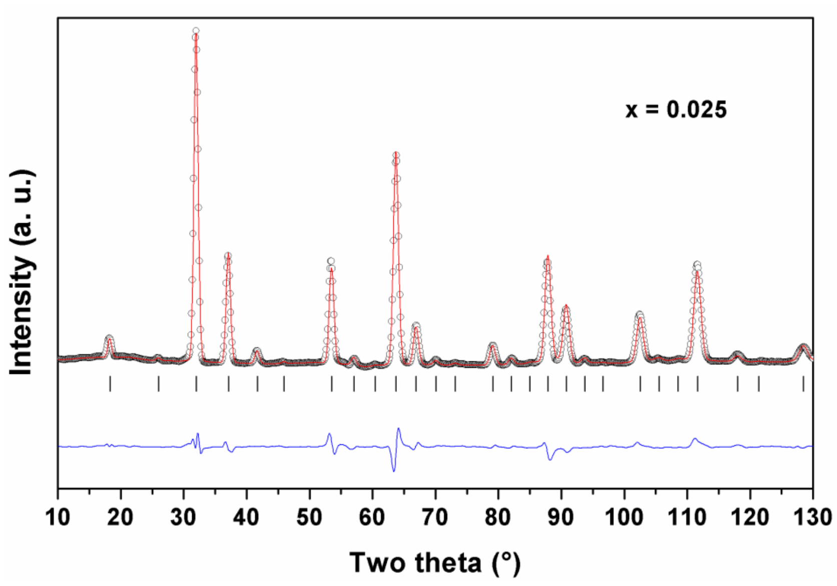

Fig. S-I(b). Rietveld refinement plot for x = 0.05 ($SrTi_{0.95}Fe_{0.025}Ta_{0.025}O_3$). Cubic (Pm3m)
(vertical ticks for Bragg position)

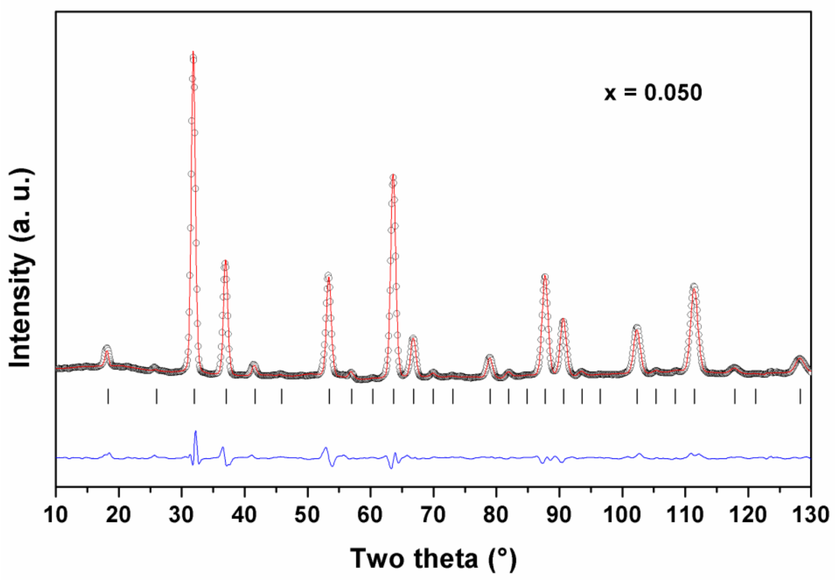

Fig. S-I(c).Rietveld refinement plot for x = 0.05 ($SrTi_{0.90}Fe_{0.05}Ta_{0.05}O_3$). Cubic (Pm3m) (vertical ticks for Bragg position).

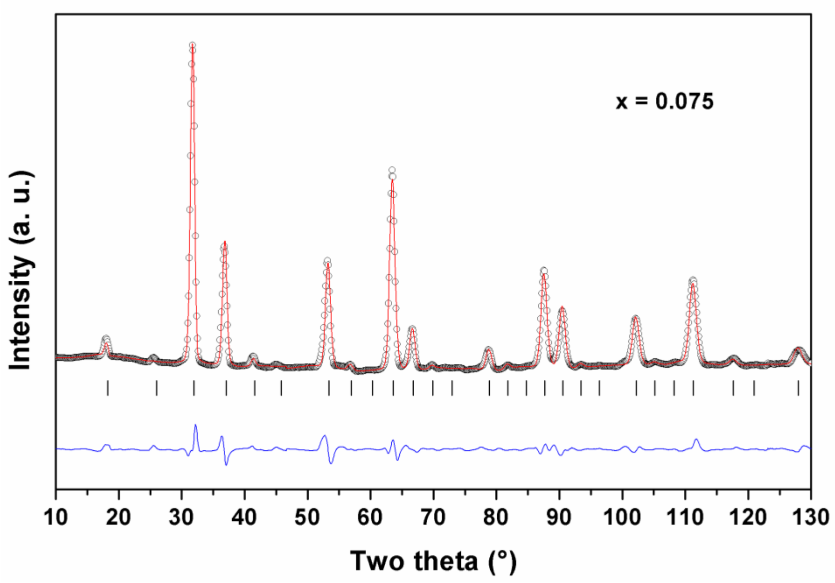

Fig. S-I(d).Rietveld refinement plot for x = 0.075 ($SrTi_{0.85}Fe_{0.075}Ta_{0.075}O_3$). Cubic (Pm3m) (vertical ticks for Bragg position)

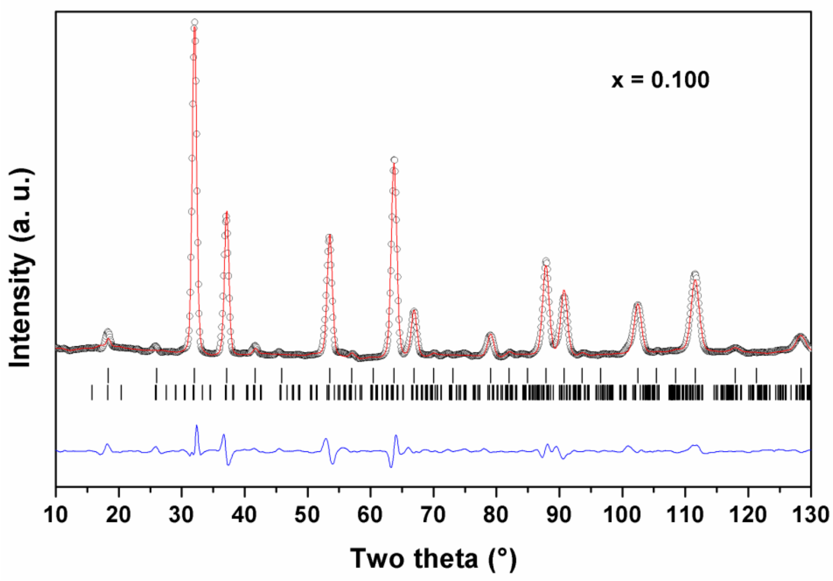

Fig. S-I(e).Rietveld refinement plot for x = 0.10 ($SrTi_{0.80}Fe_{0.10}Ta_{0.10}O_3$)
(vertical ticks for Bragg position: Cubic (upper) and Orthorhombic (lower))

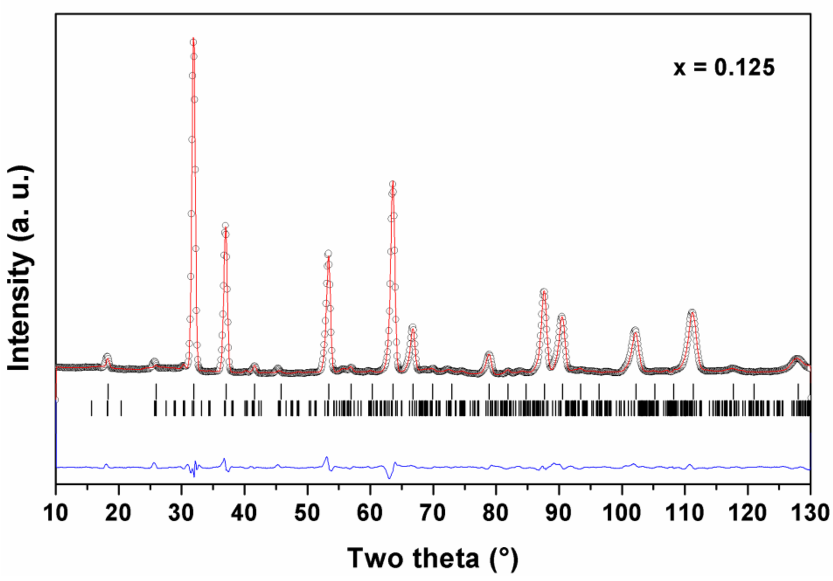

Fig. S-I(f).Rietveld refinement plot for x = 0.125 ($SrTi_{0.75}Fe_{0.125}Ta_{0.125}O_3$),
(vertical ticks for Bragg position: Cubic (upper) and Orthorhombic (lower))

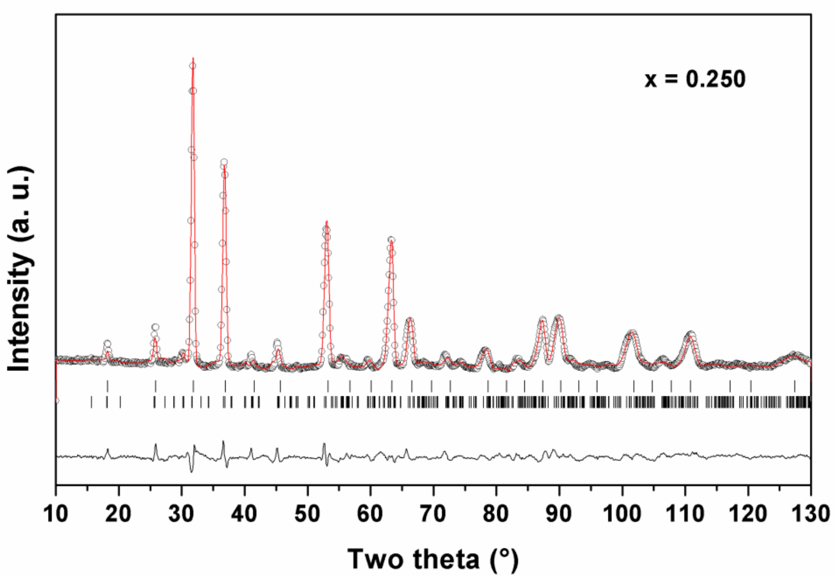

Fig. S-I(g).Rietveld refinement plot for x = 0.25 ($SrTi_{0.50}Fe_{0.25}Ta_{0.25}O_3$), (vertical ticks for Bragg position: Cubic (upper) and Orthorhombic (lower))

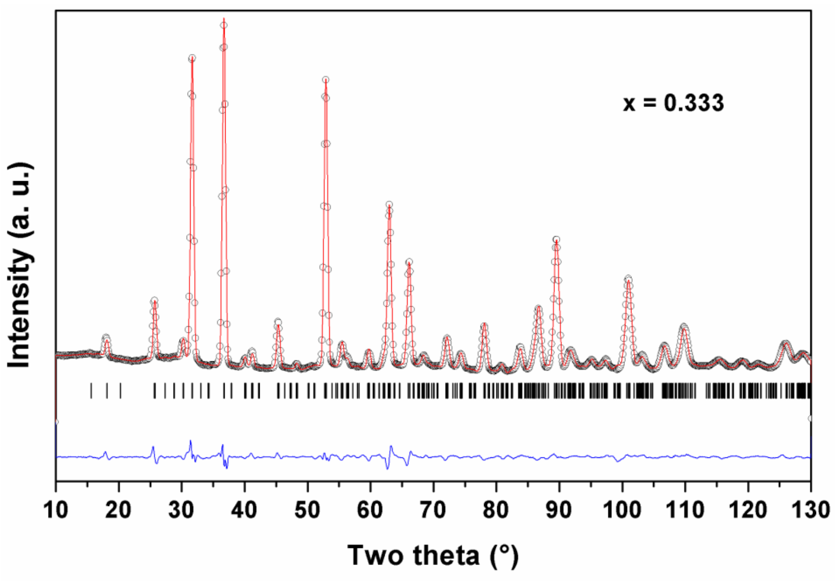

Fig. S-I(h).Rietveld refinement plot for x = 0.333 ($SrTi_{0.333}Fe_{0.333}Ta_{0.333}O_3$): Orthorhombic (Pbnm) (vertical ticks for Bragg position)

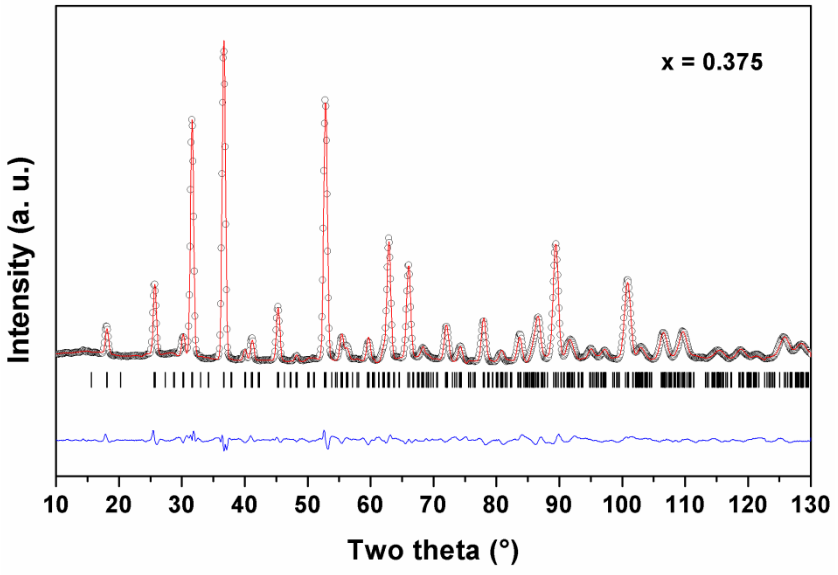

Fig. S-I(i).Rietveld refinement plot for x = 0.375 ($SrTi_{0.25}Fe_{0.375}Ta_{0.375}O_3$): Orthorhombic (Pbnm) (vertical ticks for Bragg position)

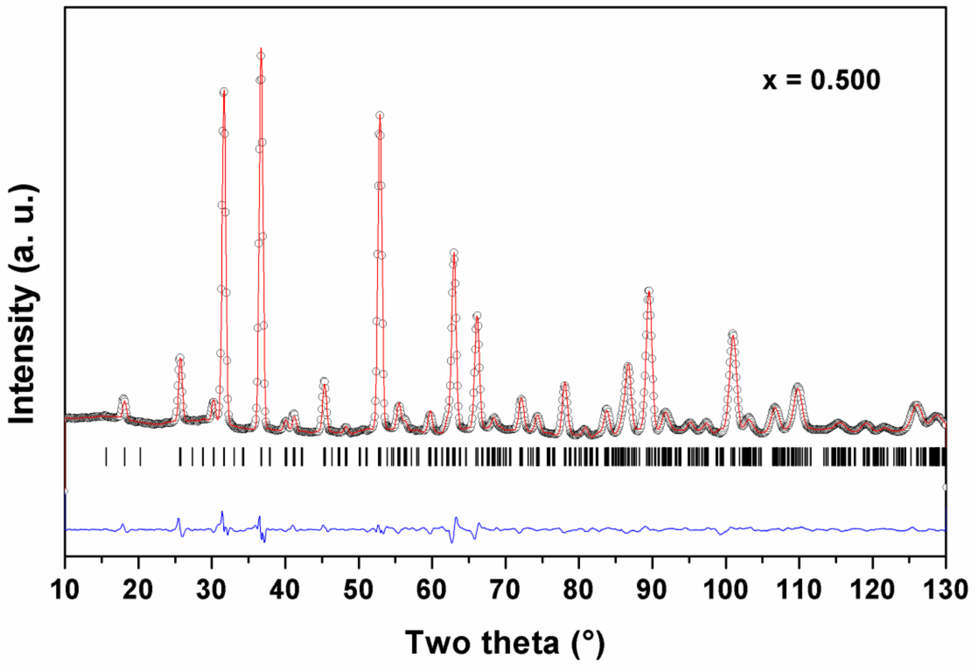

Fig. S-I(j).Rietveld refinement plot for x = 0.50 ($SrTi_{0.00}Fe_{0.50}Ta_{0.50}O_3$): Orthorhombic (Pbnm) (vertical ticks for Bragg position)

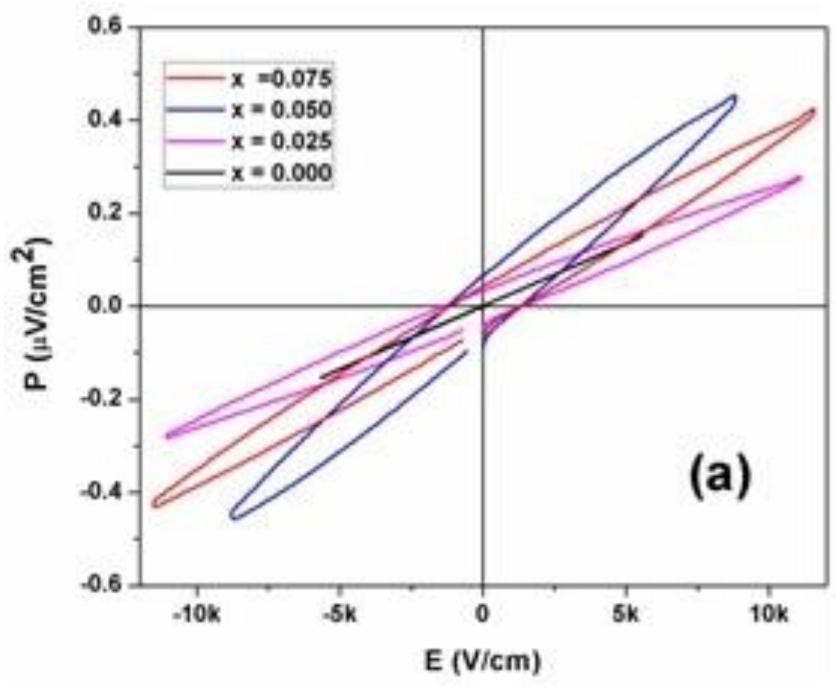

Figure S-II**:** Polarization vs Electric field loops of cubic solid solution phases of $SrTi_{1-2x}Fe_xTa_xO_3$ ($0.00 \le x \le 0.075$) compositions. Measured at the frequency 150 Hz and at ambient temperature by using Aixacct TF2000 (Aixacct GmbH, Germany) ferroelectric analyzer.
